# Outcomes of care programme approach, dual diagnosis, carer support and psychological therapy inductions

**DOI:** 10.1192/pb.bp.113.045526

**Published:** 2014-08

**Authors:** Caroline Kamau

**Affiliations:** 1 Birkbeck, University of London

## Abstract

**Aims and method** For many trainees, an obstacle into psychiatry is the challenge of an imprecise job design and uncertainty about the psychiatrist’s job design across many complex, often *ad hoc* care situations involving multiple professions and organisations. The UK’s National Health Service (NHS) has introduced inductions for trainee psychiatrists geared towards improving that. Are the induction programmes effective? This article presents an analysis of the outcomes (*n* = 1115) of inductions about the care programme approach, dual diagnosis, carer support, mental health risk assessment, psychological therapy and suicide risk assessment.

**Results** Univariate analyses of variance revealed a consistent interaction of care programme approach, dual diagnosis, carer support and psychological therapy inductions. Psychiatrists who attend all four inductions have the best perceptions about their job design, strongest teamwork approach, and highest motivation.

**Clinical implications** The NHS and hospitals outside the UK should note these results when prioritising inductions for trainee psychiatrists.

There is a global shortage of psychiatrists.^1^ One factor which dissuades would-be psychiatrists is uncertainty about the job design, relative to other areas of medicine. Psychiatric practice in healthcare systems such as the UK’s National Health Service (NHS) involves a complex multiprofessional and multi-organisational network and this creates job role uncertainty for trainees who have not been well inducted. Psychiatrists writing in the early 1990s called for better provision of induction programmes,^2^ as only 68% were being provided with a comprehensive induction programme that addressed the multiprofessional aspects of psychiatric practice.^3^ Health systems globally have since increased their spending on human resource development; for instance, the NHS has outlined a new policy framework which encourages training, professional development, lifelong learning and induction programmes to increase staff motivation, generate positive impressions of the organisation, facilitate team development, and help staff learn about their job role.^4,5^ Is this working in psychiatry? Questions have been raised before about the usefulness of some of the induction programmes used within the NHS, such as the care programme approach induction.^6^ Over 10 years after the introduction of these induction programmes, we need a fresh analysis of their simple and combined effects. Clarifying the usefulness of different induction programmes has implications for mentors, the NHS and mental health systems in other countries, as they review the induction process for trainees.

## Method

This analysis began with an extraction of data from the NHS staff survey conducted by the Care Quality Commission and Picker Institute Europe in 2011.^7^ There were 134 967 NHS employees who responded and data were extracted from the 1115 medical doctors who completed one or more of six psychiatric inductions. These were:

- A, a care programme approach (CPA) induction
- B, a mental health risk assessment induction
- C, an induction about suicide risk assessment
- D, an induction about carer support
- E, a dual diagnosis induction
- F, an induction about psychological therapies.

The variables were recoded so that a value of ‘1’ was assigned if a doctor had completed the induction within the past 12 months or before, and ‘0’ if not. This analysis also utilised doctors’ responses to questionnaires which measured perceptions about job design, teamwork and feelings of motivation at work.

## Results

Univariate analyses of variance using SPSS/PASW 18 software for Windows calculated the main and interaction effects of the inductions as independent variables.

### Effects of inductions on perceptions of job design quality

**Fig 1:**
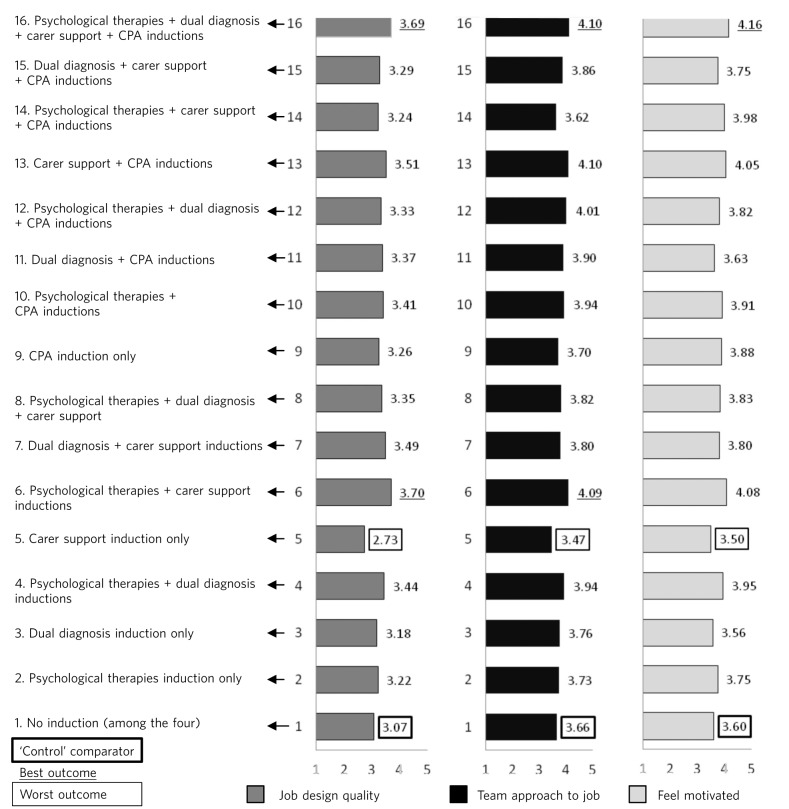
The four-way (2 × 2 × 2 × 2) interaction effect of care programme approach (CPA), dual diagnosis, carer support and psychological therapy inductions. It predicts psychiatrists’ perceptions of their job design, how much their job uses a teamwork approach, and how motivated they feel at work. The carer support induction programme by itself produces a significantly worse outcome than no induction. Combination #16 produces the best outcomes across all dependent measures.

The first univariate analysis showed the effects of the induction programmes on the psychiatrists’ appraisal of the quality of their job design. The following effects were significant, all of which were interactions: A × B × E = job design: *F*_(1,926)_ = 5.10, *P* = 0.024, η^2^ = 0.005; A × C × D = job design: *F*_(1,926)_ = 5.10, *P* = 0.024, η^2^ = 0.005; A × C × E = job design: *F*_(1,926)_ = 5.63, *P* = 0.018, η^2^ = 0.006; A × D × E = job design: *F*_(1,926)_ = 6.37, *P* = 0.012, η^2^ = 0.007; A × B × C × D = job design: *F*_(1,926)_ = 4.23, *P* = 0.039, η^2^ = 0.005; A × D × E × F = job design: *F*_(1,926)_ = 14.55, *P* = 0.000, η^2^ = 0.015. Figure 1 shows the pattern of means within the A × D × E × F interaction, which had the largest effect size (η^2^).

### Effects of inductions on appraisal of teamwork

The next univariate analysis of variance showed the effects of the induction programmes on psychiatrists’ appraisal of their teamwork and its quality, including team members having shared objectives, team members meeting to discuss team effectiveness, and team members communicating closely. The significant effects were as follows, all of which were interactions: A × B = teamwork: *F*_(1,916)_ = 3.80, *P* = 0.05, η^2^ = 0.004; B × F = teamwork: *F*_(1,916)_ = 4.73, *P* = 0.03, η^2^ = 0.005; A × D × E = teamwork: *F*_(1,916)_ = 3.95, *P* = 0.047, η^2^ = 0.004; B × E × F = teamwork: *F*_(1,916)_ = 4.46, *P* = 0.035, η^2^ = 0.005; C × E × F = teamwork: *F*_(1,916)_ = 4.06, *P* = 0.004, η^2^ = 0.004; A × D × E × F = teamwork: *F*_(1,916)_ = 7.05, *P* = 0.008, η^2^ = 0.008. This four-way interaction replicated the results of the first univariate model which predicted the psychiatrists’ perceptions about the quality of their job design, also showing that this interaction had the largest effect size. Fig. 1, showing the pattern of means within the interaction A × D × E × F, shows a mirroring of the pattern from the model which predicted perceptions about job design.

### Effects of inductions on feeling motivated

The third univariate analysis of variance showed the effects of the induction programmes on the psychiatrists’ feelings of motivation at work. The following were significant effects, all interactions: A × E = feel motivated: *F*_(1,932)_ = 3.76, *P* = 0.05, η^2^ = 0.004; C × D × F = feel motivated: *F*_(1,932)_ = 4.10, *P* = 0.04, η^2^ = 0.004; A × D × E × F = feel motivated: *F*_(1,932)_ = 4.09, *P* = 004, η^2^ = 0.004. Therefore, the four-way interaction of inductions A, D, E and F found in the first two models was replicated. As Fig. 1 shows, the pattern of means mirrored that from the first and second univariate analyses.

In summary, the combination of induction programmes with consistently good outcomes, noting the significance of an interaction across different outcomes and the effect size, is A × D × E × F. This comprises the CPA induction, carer support induction, dual diagnosis induction and psychological therapies induction. Looking at Fig. 1, with the exception of a carer support induction by itself, comparing the control group outcomes with the other conditions shows that an induction or a combination of inductions produces better outcomes than no induction.

## Discussion

This is the first analysis of the effectiveness of induction programmes for psychiatrists. The most consistently effective combination, across three outcomes, comprises the CPA induction, the carer support induction, the dual diagnosis induction and the psychological therapies induction. The three outcomes are: the psychiatrists’ perceptions of the quality of their job design, the quality of teamwork and their motivation at work. These four induction programmes give trainee psychiatrists realistic previews of their job role and workplace, boosting the positive outcomes. This finding extends robust empirical evidence about the worth of realistic job previews and job role unambiguity^8,9^ in the context of psychiatric training. One meta-analysis of 52 studies found that realistic job previews increase employees’ perceptions of their job role clarity.^8^ Another meta-analysis of studies totalling 35 265 employees found that ambiguity about one’s job role is one of the most serious work stressors that has an impact on job performance.^9^

Further research should explore the reasons for non-uptake of each induction programme. One reason is a lack of access, and another reason is a trainee’s initial level of motivation. Trainees who present a high level of motivation might be more likely to take up an induction than other trainees. A follow-up study should therefore use an experimental design and code the element of choice as an independent variable, as well as measuring the trainees’ motivation before and after each induction. This would allow an analysis of both the between-subjects effects analysed in the present study and within-subjects effects not analysed. The issue of access also warrants a national audit of the availability of different induction programmes. The present results should be taken into consideration in prioritising particular inductions. This analysis has shown that a CPA induction works well only in combination with other induction programmes. Alone, it is only marginally better than a control group on three outcomes (job design, teamwork and motivation). This analysis has also revealed that the induction provision with the worst outcome is that whereby psychiatrists only attend a carer support induction. The NHS, and hospitals outside the UK, should note this evidence when planning inductions of trainees.
